# Supplementary material for: Multi-Attribute Subset Selection enables prediction of representative phenotypes across microbial populations
Source: Commun Biol. 2024 Apr 3;7:407. doi: 10.1038/s42003-024-06093-w (PMC10991586; doi:10.1038/s42003-024-06093-w)
Supplement: Supplementary file 2 — Supplementary Information [file 42003_2024_6093_MOESM2_ESM.pdf]

Supplementary Information

Prediction of representative phenotypes using Multi-Attribute Subset Selection

Konrad Herbst[1,2,*], Taiyao Wang[3,*], Elena J. Forchielli[2,4,*], Meghan Thommes[2,5,*], Ioannis Ch. Paschalidis[3,5,6,7,#], Daniel Segrè[1,2,4,5,6,#]

[1]Bioinformatics Program, Boston University, Boston, MA, USA
[2]Biological Design Center, Boston University, Boston, MA, USA
[3]Division of Systems Engineering, Boston University, Boston, MA, USA
[4]Department of Biology, Boston University, Boston, MA, USA
[5]Department of Biomedical Engineering, Boston University, Boston, MA, USA
[6]Faculty of Computing and Data Science, Boston University, Boston, MA, USA
[7]Department of Electrical and Computer Engineering, Boston University, Boston, MA, USA

* Equally contributing authors
# Corresponding authors

Supplementary Data

Supplementary Data 1:
DATASET 1 strains with additional metadata.

Supplementary Data 2:
Digitized values of table in Chapter 6 of (Barnett, Payne, and Yarrow 1990) resulting 590 yeast and 92 phenotypes (raw data for DATASET 3).

Supplementary Data 3:
Selection of attributes of **Supplementary Data 2** used for MASS application.

Supplementary Data 4:
Source Data for **Figure 2c-d, 3c-d, 4c-d** and **Supplementary Figure 2, 4, 5**.

Supplemental Figures

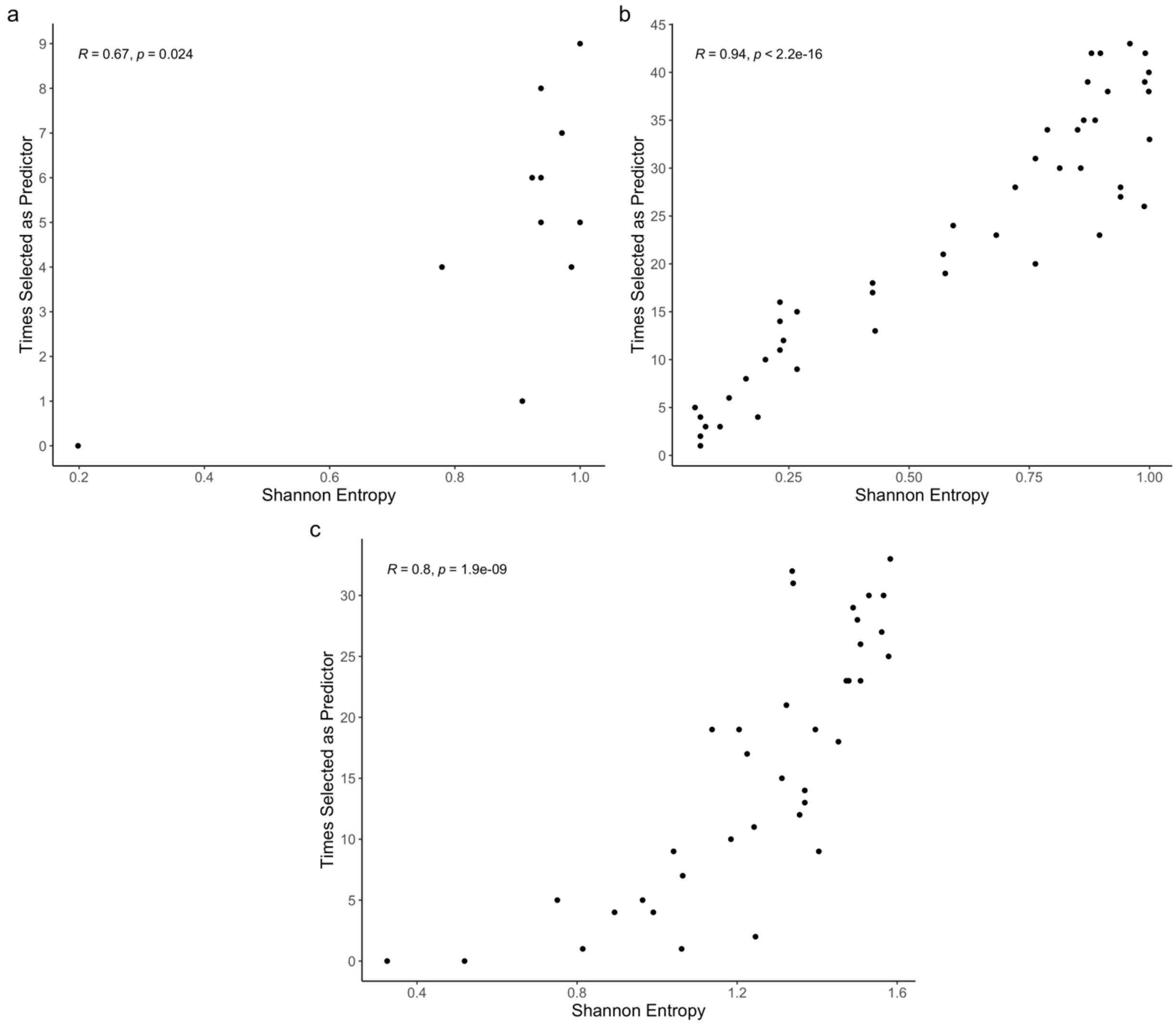

Supplementary Figure 1. The variable predictor likelihood versus its information content. The number of times a variable was selected as a predictor as a function of Shannon entropy for the marine bacterial growth DATASET 1 (**a**), the bacterial fermentation DATASET 2 (**b**), and the yeast phenotype DATASET 3 (**c**). The plots also contain the Pearson correlation coefficients for comparison.

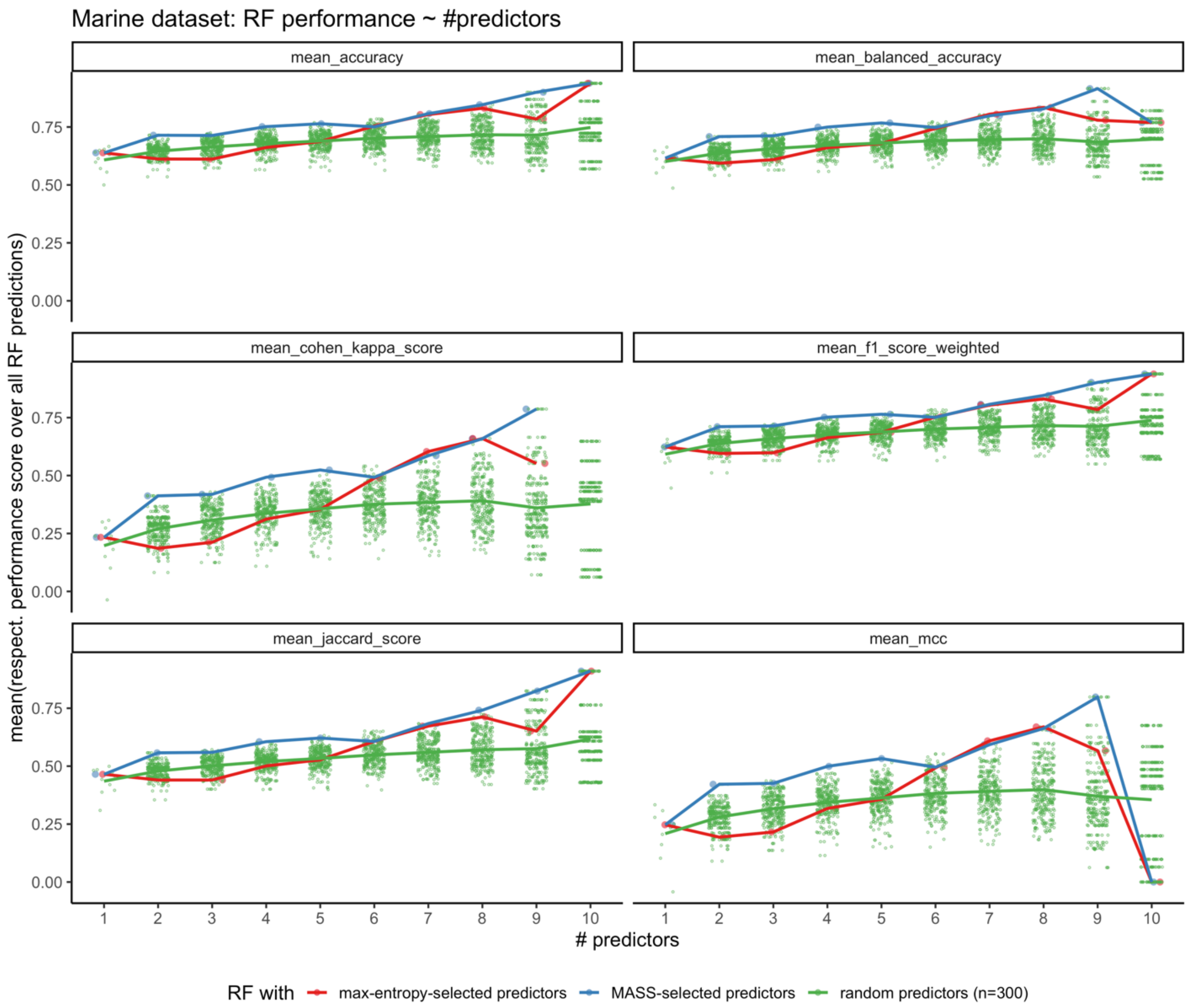

Supplementary Figure 2. Various performance measures of random forest classifiers for DATASET 1. Random forest (RF) classifiers were trained using environmental conditions selected by MASS (blue), maximal Shannon entropy (red), or random conditions as predictors. The classifiers were then evaluated using (from top-left to bottom-right panel) Accuracy, Balanced Accuracy, Cohen's Kappa score, weighted F1 score, Jaccard score and Matthews correlation coefficient (MCC). The MCC plot was used as **Figure 2d**. Source Data is available in **Supplementary Data 4**.

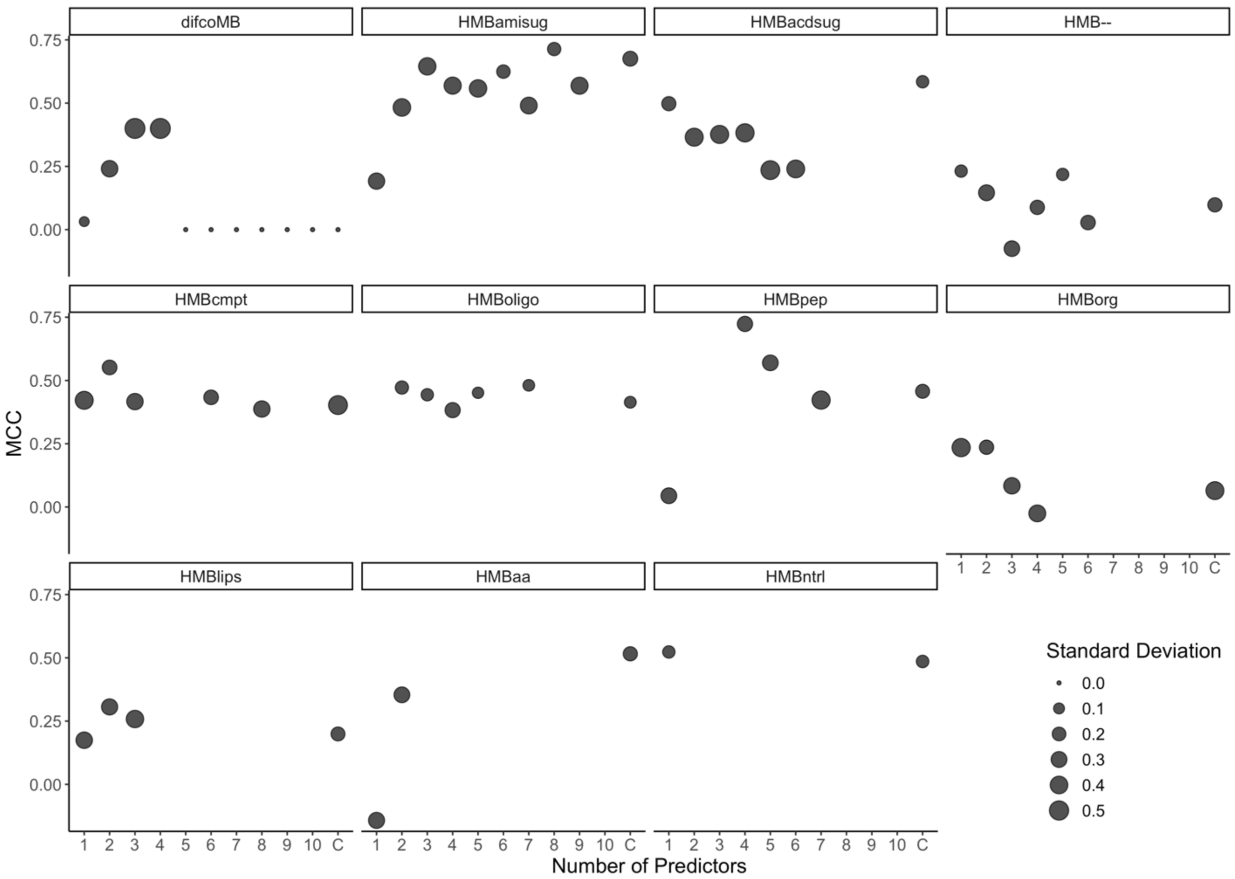

Supplementary Figure 3. DATASET 1 performance measures of random forest classifiers for individual responses. For each possible response condition, the Matthews correlation coefficient (MCC) of each random forest model for each number of predictors, *p*, is depicted. Dot size represents the standard deviation of performance scores for values obtained via 5-fold cross-validation; column C is the RF control using all other conditions as predictors to predict a response condition. If no dot is displayed for a condition, the condition was selected as a predictor. Media conditions are ordered based on their likelihood to be selected as response by MASS and named as follows: Difco Marine Broth (difcoMB), eight engineered media with single classes of carbon sources (HMBpep = peptides; HMBaa = amino acids; HMBlips = lipids; HMBoligo = oligosaccharides; HMBorg = organic acids; HMBntrl = neutral sugars; HMBamisug = amino sugars; HMBacdsug = acidic sugars), a defined medium containing all 8 carbon classes (HMBcmpt), and a medium with no added carbon sources (HMB–).

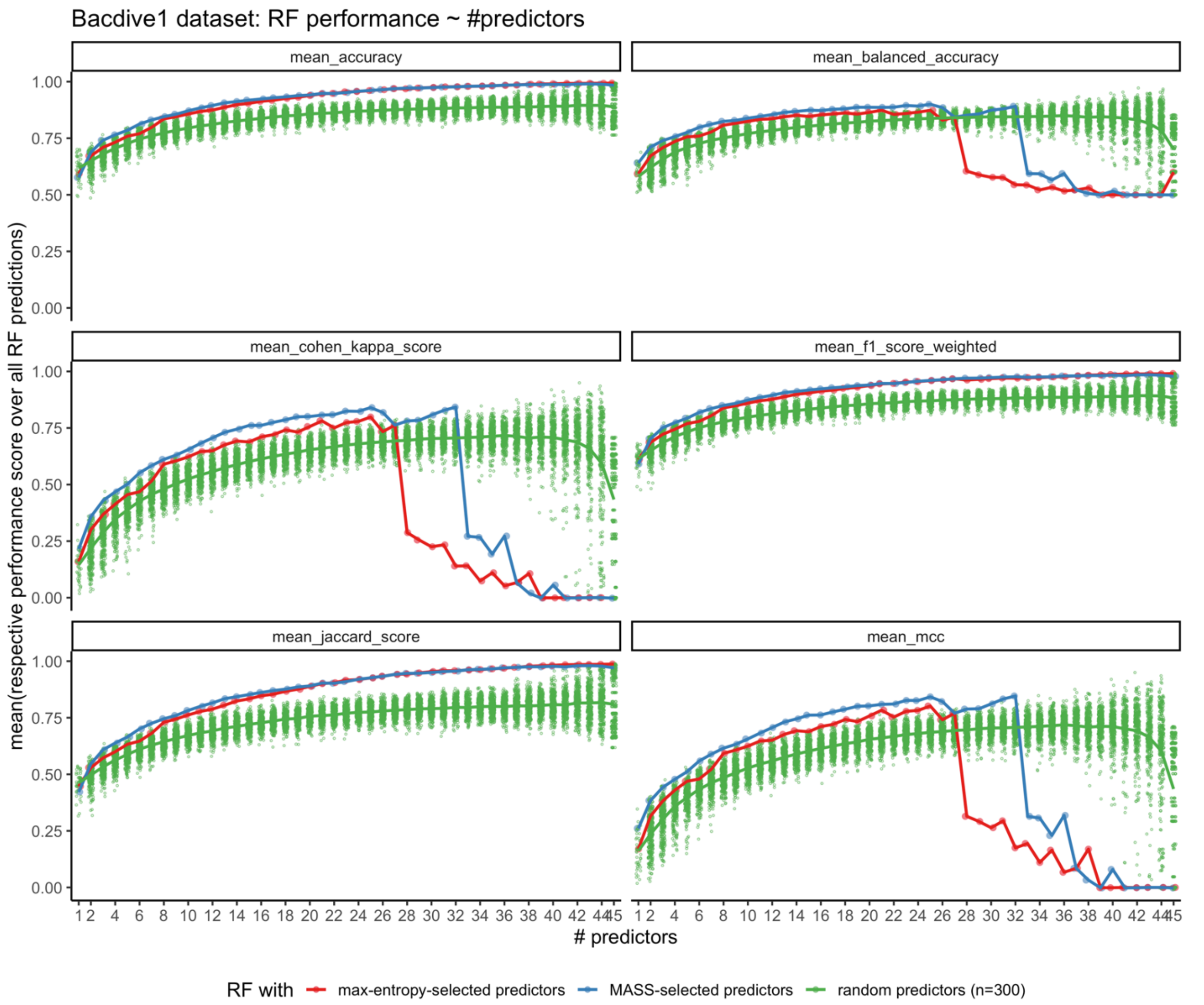

Supplementary Figure 4. Various performance measures of random forest classifiers for DATASET 2. Random forest (RF) classifiers were trained using environmental conditions selected by MASS (blue), maximal Shannon entropy (red), or random conditions as predictors. The classifiers were then evaluated using (from top-left to bottom-right panel) Accuracy, Balanced Accuracy, Cohen's Kappa score, weighted F1 score, Jaccard score and Matthews correlation coefficient (MCC). The MCC plot was used as **Figure 3d**. Source Data is available in **Supplementary Data 4**.

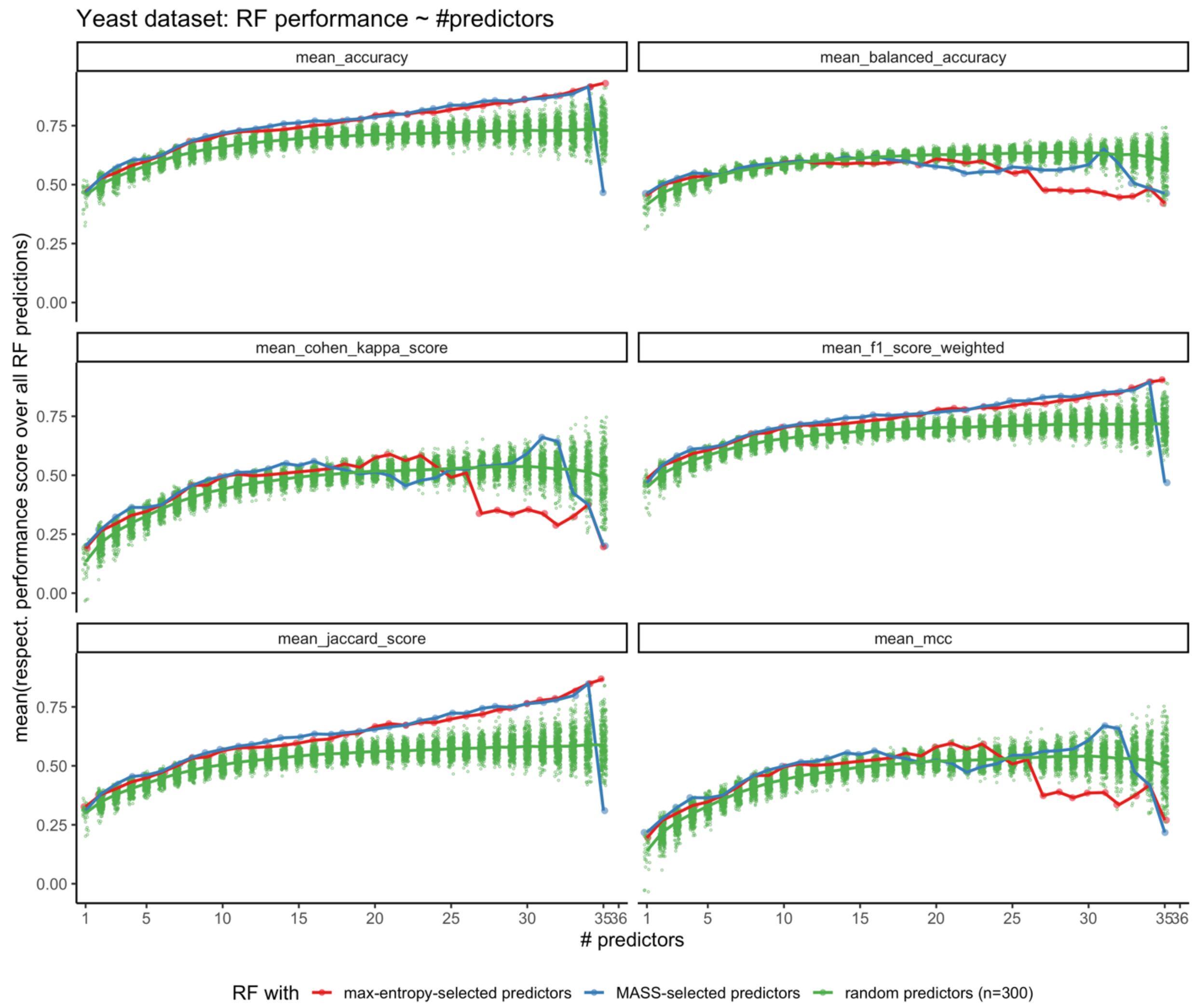

Supplementary Figure 5. Various performance measures of random forest classifiers for DATASET 3. Random forest (RF) classifiers were trained using environmental conditions selected by MASS (blue), maximal Shannon entropy (red), or random conditions as predictors. The classifiers were then evaluated using (from top-left to bottom-right panel) Accuracy, Balanced Accuracy, Cohen's Kappa score, weighted F1 score, Jaccard score and Matthews correlation coefficient (MCC). The MCC plot was used as **Figure 4d**. Source Data is available in **Supplementary Data 4**.
