# Supplementary material for: Multi-Attribute Subset Selection enables prediction of representative phenotypes across microbial populations
Source: Commun Biol. 2024 Apr 3;7:407. doi: 10.1038/s42003-024-06093-w (PMC10991586; doi:10.1038/s42003-024-06093-w)
Supplement: Supplementary file 3 — Description of Supplementary Materials [file 42003_2024_6093_MOESM3_ESM.docx]

**Description of Additional Supplementary Files**

**File name:** Supplementary Data 1

**Description:** DATASET 1 strains with additional metadata

**File name:** Supplementary Data 2

**Description:** Digitized values of table in Chapter 6 of (Barnett, Payne, and Yarrow 1990) resulting 590 yeast and 92 phenotypes (raw data for DATASET 3).

**File name:** Supplementary Data 3

**Description:** Selection of attributes of Supplementary Data 2 used for MASS application.

**File name:** Supplementary Data 4

**Description:** Source Data for Figure 2c-d, 3c-d, 4c-d and Supplementary Figure 2, 4, 5.
